# *EyeChrom* and *CCDBcurator:* Visualizing chromosome count data from plants

**DOI:** 10.1002/aps3.1207

**Published:** 2019-01-04

**Authors:** Rodrigo Rivero, Emily B. Sessa, Rosana Zenil‐Ferguson

**Affiliations:** ^1^ Department of Biology University of Florida Box 118525 Gainesville Florida 32611 USA; ^2^ Department of Natural Resources and Environmental Management University of Hawaii 1910 East‐West Road Manoa Hawaii 96822 USA; ^3^ Department of Ecology, Evolution, and Behavior University of Minnesota 1479 Gortner Avenue St. Paul Minnesota 55108 USA

**Keywords:** chromosome, data visualization, genome size, karyotype, polyploidy

## Abstract

**Premise of the Study:**

Chromosome count data are available for hundreds of plant species and can be explored in text‐only format at the Chromosome Counts Database (http://ccdb.tau.ac.il). *CCDBcurator* and *EyeChrom* are an R package and a web application, respectively, that first curate and then visualize these data graphically, so that intra‐ and interspecific variation of chromosome numbers can be easily summarized and displayed for a given genus.

**Methods and Results:**

We developed R code to clean, summarize, and display in several formats the chromosome count data for a selected genus or set of species present in the Chromosome Counts Database. These data and figures can be exported for use in analyses, publications, or teaching.

**Conclusions:**

Chromosome count data are critical for a number of evolutionary studies in plant biology, and their importance is underscored by the increasing appreciation of the prevalence of polyploidy in land plants. *CCDBcurator* and *EyeChrom* provide a fast, easy, and reproducible means of cleaning, curating, and then visualizing the chromosome count data currently available for plants.

Analyses of plant chromosome numbers have been used for decades to understand relationships among plants and, more recently, to assess patterns of genome evolution. Visualization of karyotypes (the chromosome complement of an organism) beginning in the 1800s allowed counting as well as assessment of the appearance of chromosomes. Works like Irene Manton's *Problems of Cytology and Evolution in the Pteridophyta* (Manton, 1950) contributed substantially to the numbers of published chromosome counts for plants. These data have facilitated insights on plant evolution and the prevalence of polyploidy in plants in addition to untangling species complexes (Williams, 2016).

Polyploidy, or whole genome duplication, is a common evolutionary phenomenon that is particularly prevalent in plants (Wood et al., 2009) and that generates individuals with extra sets of chromosomes. Polyploidy can occur after the union of two gametes that are “unreduced” and therefore diploid, as opposed to the haploid gametes that would typically result from normal meiosis; syngamy of such unreduced gametes produces a zygote with more than the standard two sets of chromosomes (i.e., instead of one set from each parent, there are two or more sets from each, depending on the ploidy levels of the parents) (Harlan and DeWet, 1975; Brownfield and Köhler, 2011). Polyploidy can also occur via a “triploid bridge,” in which union of one reduced and one unreduced gamete produces a (typically sterile) triploid that then undergoes whole genome duplication, resulting in a polyploid organism with proper chromosome pairing behavior, and therefore fertility, restored (Harlan and DeWet, 1975; Ramsey and Schemske, 1998). Polyploid organisms are often classified as either autopolyploid or allopolyploid depending on whether the progenitor taxa belong to the same or different species, respectively, although these two terms have long been recognized as ends of a continuum rather than a hard dichotomy (Stebbins, 1971; Soltis et al., 2014).

Polyploidy is now seen as an important process in plant diversification that has occurred throughout land plant evolution (Soltis et al., 2015; Wendel, 2015). Evolutionary biologists have studied the effects of polyploidy on myriad aspects of plant evolution, including genome structure, epigenetics, ecology, fitness, invasive potential, pollinator interactions, and ability to establish and diversify (Thompson et al., 2004; Thompson and Merg, 2008; te Beest et al., 2012; Madlung, 2013; Madlung and Wendel, 2013; Weiss‐Schneeweiss et al., 2013; Parisod and Broennimann, 2016; Van de Peer et al., 2017) (see also the *American Journal of Botany* special issue: The Evolutionary Importance of Polyploidy, e.g., Ågren et al., 2016; Husband et al., 2016; Segraves and Anneberg, 2016; Zenil‐Ferguson et al., 2016). The basic establishment of whether or not a species is polyploid, however, still comes down to chromosome count data, which for more than 50 years were only available in books (Manton, 1950; Darlington and Wylie, 1955) or in databases that did not aggregate all genera (Goldblatt and Lowry, 2011). Although methods like flow cytometry (Dolezel and Bartos, 2005) and sequencing‐based approaches (Vurture et al., 2017) are now widely used to estimate genome sizes and predict ploidy levels, these methods must ultimately rely on comparisons to chromosome numbers previously obtained from counts, either newly collected or in most cases from past literature. Chromosome count data therefore remain a critical information source for plant biologists studying polyploidy and genome size evolution.

Currently, the largest source of plant chromosome count information available is the Chromosome Counts Database (CCDB; http://ccdb.tau.ac.il/), which is a regularly updated repository of chromosome counts drawn from numerous sources in the literature (Rice et al., 2015). The CCDB displays in tabular format the chromosome count mode available for a given species, as well as the original source reference and records for that species, and whether each count is gametophytic (*n*) or sporophytic (2*n*). These full records are easily exportable to CSV format directly from the CCDB website or via the R package *chromer* (Pennell, 2016). However, the data displayed in the CCDB and that are available for export are raw and uncurated, which can be problematic if the raw counts are formatted differently or are represented using different patterns. In addition, the downloadable CSV file will provide a parsed value for each entry in the data set that has a non‐numeric character format, and this parsing is accomplished using rules that are not transparent or reproducible. Therefore, the export tools available directly from the CCDB cannot provide a comprehensive list of chromosome numbers for multiple species simultaneously. This is a serious limitation, as interspecific and intergeneric chromosomal variation are key for the development of models in polyploidy research, among other things (Zenil‐Ferguson et al., 2016, 2017). For example, if bivalents as well as trivalents or quadrivalents were visible in a karyotype, historically this was represented using notation that indicates the number of each type seen. For example, *Blechnum occidentale* L. has one count in the CCDB recorded as “40II+44I,” which means that 40 bivalents were present (each bivalent is a pair of chromosomes, indicated by the “II” after the 40) in addition to 44 univalents (singleton chromosomes, indicated by the “I” after the 44). The actual count is therefore 40 + 40 + 44 = 124 chromosomes, which suggests that this individual may have been a tetraploid (the base number in *Blechnum* is *x* = 31; Gasper et al., 2016). In the downloadable CSV version of the CCDB data, this same record shows as “40II+44I” and is parsed as “40,” losing critical information about the complete chromosome complement. Entries in the database can differ in their formats in other ways as well (Table 1), resulting in additional opportunities for information loss or mishandling.

**Table 1:** Examples of count translations (i.e., curated, clean records) produced via the *CCDBcurator* R package. Original records in the CCDB include thousands of different patterns, which makes accurate interpretation of chromosome numbers challenging. *CCDBcurator* cleans the most common patterns in the original records using perl‐like regular expressions. These clean records become the input for visualization in *EyeChrom* and are downloadable for quantitative analyses or further cleaning. Users can report cleaning issues or suspected new patterns to https://github.com/roszenil/CCDBcurator

Taxon	Count type	Original record format (exact text from CCDB)	*CCDBcurator* count translation
*Gomphrena globosa*	Sporophytic	40‐44	40 44
*Barnardia japonica*	Sporophytic	34+0‐13,16,27,30f,etc	34 16 27 30
*Gentiana terglouensis*	Sporophytic	11II+16I;19II	38 38
*Chrysanthemum morifolium*	Sporophytic	62+1B, 62+2Bs, 63, 63+1B, 64(1, 1, 1, 4, 1, 1)	62 62 63 63 64
*Heracleum sphondylium*	Sporophytic	11II+1BI	22

Extracting and summarizing uncurated or incomplete data from the CCDB has been accomplished previously by software packages including *chromer*, but creating a uniformly curated version of these data that is easily accessible is challenging given the number of data patterns present in the uncurated data. (There are thousands of these patterns in the CCDB; the most commonly encountered are given in Table 1.) Furthermore, data sets cleaned by individual researchers are not available via a public‐facing application (i.e., one that requires no coding skills). Here we present a web application (*EyeChrom*) and an R package (*CCDBcurator*) that together allow for comprehensive, reproducible curation and visualization of CCDB chromosome count data. The first complete version of the R package *CCDBcurator* is documented here (it was first described as a set of scripts by Zenil‐Ferguson et al. 2017)*. EyeChrom* visualizes the processed data from *CCDBcurator* for a given genus or set of species using both plots and tables, and allows these curated data to be exported for further cleaning or analysis, including the original records reported by CCDB for comparison.

Variation in chromosome counts for a genus or even a species can be difficult to visualize and summarize, and presenting this information in an easily accessible, graphical format for many taxa simultaneously should be a useful tool for researchers as well as instructors interested in teaching about chromosomes, karyotypes, polyploidy, or genetics. Visualization also allows easy detection of mistakes in pattern curation, allowing users to quickly identify and report new patterns of chromosome numbers in the original database that are not captured in our curation code and need to be incorporated, allowing the R package and web application to work in tandem to visualize and improve curation of chromosome count data (Fig. 1). The *EyeChrom* web application aims to accomplish these goals by using simple drop‐down controls and menus, being web‐based, accessible from any browser, and available at no cost, and therefore approachable for users not comfortable with programming languages. Simultaneously, users that are familiar with R programming can access the curated records via *CCDBcurator*, clean and upload their own data, and dynamically contribute to the improvement of pattern detection via GitHub.

**Figure 1:**
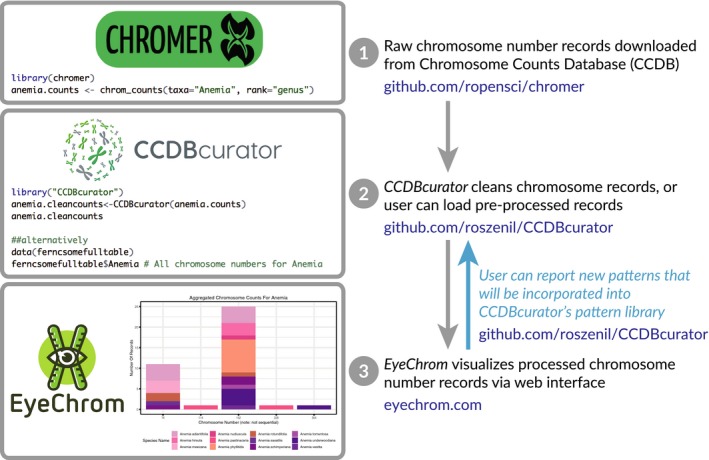
Diagram showing the workflow for *CCDBcurator* and *EyeChrom*. Steps 1 and 2 are completed automatically, and the curated data are visualized via *EyeChrom* in Step 3. Step 1: CCDB records are first obtained using the R package *chromer* (Pennell, 2016). Step 2: *CCDBcurator* cleans the records and prepares a data frame in the format used by *EyeChrom*. Alternatively, users can upload pre‐processed data frames. Step 3: Users visualize records online using the *EyeChrom* interface (available at http://eyechrom.com) and can report potential issues in the count patterns and translation via https://github.com/roszenil/CCDBcurator or https://github.com/RodrigoRivero/EyeChrom. Users can also improve the pattern recognition by cloning the GitHub repositories of *CCDBcurator* and *EyeChrom*.

## METHODS AND RESULTS

### Data source and processing

The CCDB (http://ccdb.tau.ac.il/) (Rice et al., 2015) is the source of primary data for *EyeChrom*. *EyeChrom* uses a version of the CCDB database that has first been processed/curated using tools in the R package *CCDBcurator*. The output of *CCDBcurator* is a database free of non‐integer entries. Entries formatted using the notation described above (“40II+44I”) or that include other signs or symbols (e.g., “+”) are converted to the appropriate integer value. *CCDBcurator* recognizes the most common patterns encountered in the CCDB and cleans the records via perl‐like regular expressions, although additional patterns may exist that it does not yet recognize. *CCDBcurator* is regularly updated to incorporate these, and researchers are encouraged to submit pattern recognition updates via GitHub (https://github.com/roszenil/CCDBcurator). Examples of the types of regular expressions cleaned by *CCDBcurator* are shown in Table 1.

### Description of the application

**Figure 2:**
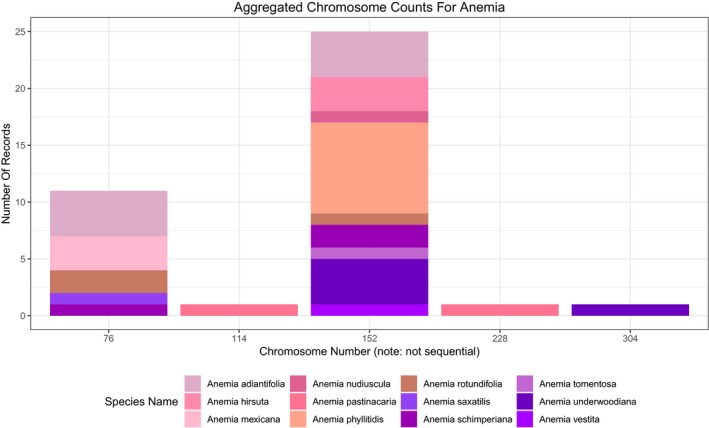
Example output of a search in *EyeChrom* for the fern genus *Anemia*. Columns in the stacked bar plot correspond to the number of curated records in the Chromosome Counts Database (CCDB) at a particular chromosome number, and are colored according to individual species. If a given species has records present at multiple chromosome numbers, it will appear in multiple bars (e.g., *Anemia adiantifolia* has counts of both 76 and 152).

Once the CCDB records are curated, the *EyeChrom* scripts ingest the curated data in a simple table format that includes taxon name, curated chromosome number, the translation made by *CCDBcurator*, and the original record as found in the CCDB. At the *EyeChrom* web application (which can be accessed at http://eyechrom.com), the user first selects the taxon group (ferns or angiosperms) and whether to display gametophytic (*n*), sporophytic (2*n*), or combined (both gametophytic and sporophytic) counts. The user then indicates the taxa they are interested in by selecting a genus from a drop‐down menu (text can also be entered into this box) and then checking or unchecking the species to be included (a “select all/deselect all” option is available). The data are then presented in several formats in separate tabs of the application, as follows: (1) A stacked bar plot, where the height of each bar corresponds to the frequency of CCDB records that have a particular count for each species selected. If more than one species (or an entire genus) was selected, each stacked bar is divided by species, which are represented by different colors, and a legend is provided to identify the species in the plot (Fig. 2). (2) An interactive plot identical to the stacked bar plot, but where the user can hover the cursor over each color block to view information about the species, chromosome number, and number of records represented by that section of the stacked bar. (3) A heatmap with cell colors corresponding to the percentage of records with a given count for the taxa selected. (4) A table showing the species name, chromosome number, original count, translated count, and whether the count is gametophytic or sporophytic.

It is not uncommon for individual species to have multiple records at different chromosome count values, which may represent polyploid series or an unidentified case of autopolyploidy. For example, the records for the fern genus *Anemia* Sw. (Anemiaceae, Schizeales) show that *A. adiantifolia* (L.) Sw. and *A. schimperiana* C. Presl (and several other taxa) have both been recorded as having 76 and 152 chromosomes (Fig. 2). These are easy to see as the same color block appearing in multiple columns in the stacked bar plot, interactive plot, and heatmap. Genera that contain potentially polyploid species can also be easily identified; *Anemia*, for example, appears to include diploid (2*n* = 2*x* = 76), tetraploid (2*n* = 4*x* = 156), hexaploid (2*n* = 6*x* = 228), and octoploid (2*n* = 8*x* = 304) species (Fig. 2).

*CCDBcurator* and *EyeChrom* were both written in R (R Development Core Team, 2016), and all graphics in *EyeChrom* are produced using the package *ggplot2* (Wickham, 2009). The web application was implemented with *Rshiny* (RStudio Inc., 2014), an R package produced by RStudio (Boston, Massachussetts, USA) for creating web applications in the RStudio In‐Development Environment (IDE). *Rshiny* makes it easy to process information and display it on a website, without the need to write in html or CSS. It also allows the creation of dynamic applications that can respond to user's selections, and has the capability of exporting data in table or text format. The application can also be run on a personal computer by cloning the *EyeChrom* repository from GitHub (https://github.com/RodrigoRivero/EyeChrom).

## CONCLUSIONS

*EyeChrom* presents information on chromosome counts from the CCDB that have been curated with *CCDBcurator* so that users can visualize these data and export them for downstream analyses. The combination of the data curation package and web application described here allows users to quickly visualize chromosome count data from the CCDB, view the distribution of these counts for taxa of interest, and assess whether genera or species are likely to include polyploids.

## AUTHOR CONTRIBUTIONS

R.Z.F. wrote the *CCDBcurator* code and conceived the application that would become *EyeChrom*; R.R. wrote the *EyeChrom* code, implemented the *Shiny* web application, and outlined the manuscript; E.B.S. consulted at all steps and oversaw manuscript preparation. All authors contributed to and approved the final version of the manuscript.

## DATA ACCESSIBILITY

*EyeChrom* is available under the GNU General Public License at https://github.com/RodrigoRivero/EyeChrom. *EyeChrom* is implemented in R and interactive online at http://eyechrom.com. *CCDBcurator* is available under the GNU General Public License at https://github.com/roszenil/CCDBcurator.
